# Realizing the measure-device-independent quantum-key-distribution with passive heralded-single photon sources

**DOI:** 10.1038/srep35394

**Published:** 2016-10-19

**Authors:** Qin Wang, Xing-Yu Zhou, Guang-Can Guo

**Affiliations:** 1 Institute of Signal Processing Transmission, Nanjing University of Posts and Telecommunications, Nanjing 210003, China; 2 Key Lab of Broadband Wireless Communication and Sensor Network Technology, Nanjing University of Posts and Telecommunications, Ministry of Education, Nanjing 210003, China; 3 Key Laboratory of Quantum Information, University of Science and Technology of China, Hefei 230026, China

## Abstract

In this paper, we put forward a new approach towards realizing measurement-device-independent quantum key distribution with passive heralded single-photon sources. In this approach, both Alice and Bob prepare the parametric down-conversion source, where the heralding photons are labeled according to different types of clicks from the local detectors, and the heralded ones can correspondingly be marked with different tags at the receiver’s side. Then one can obtain four sets of data through using only one-intensity of pump light by observing different kinds of clicks of local detectors. By employing the newest formulae to do parameter estimation, we could achieve very precise prediction for the two-single-photon pulse contribution. Furthermore, by carrying out corresponding numerical simulations, we compare the new method with other practical schemes of measurement-device-independent quantum key distribution. We demonstrate that our new proposed passive scheme can exhibit remarkable improvement over the conventional three-intensity decoy-state measurement-device-independent quantum key distribution with either heralded single-photon sources or weak coherent sources. Besides, it does not need intensity modulation and can thus diminish source-error defects existing in several other active decoy-state methods. Therefore, if taking intensity modulating errors into account, our new method will show even more brilliant performance.

The quantum key distribution (QKD) allows two legitimate users, usually called Alice and Bob, to share the secure cryptographic keys even at the existence of a malicious eavesdropper, Eve^1^. In principle, QKD can offer unconditional security guaranteed by the law of quantum physics^2^,^3^,^4^. However, due to existing imperfections in real-life QKD devices, Eve can take advantage of those loopholes and hack present QKD systems. For instance, under the circumstances of imperfect light sources, Eve can carry out the so-called photon-number-splitting (PNS) attack^5^,^6^,^7^. Fortunately, the decoy-state method was proposed to counter the PNS attack^8^,^9^,^10^, dramatically improving the performance of practical QKD system^11^,^12^,^13^,^14^. Moreover, in order to countermeasure all the potential attacks directed on the detection devices, the measurement-device-independent quantum-key-distribution (MDI-QKD) protocols were proposed^15^,^16^, which seems very promising in the implementations of QKD^17^,^18^,^19^,^20^,^21^,^22^,^23^,^24^,^25^,^26^,^27^.

During the past few years, the MDI-QKD has been widely investigated using either the heralded-single photon sources (HSPS) or the weak coherent sources (WCS). By applying different number of decoy states, all of them can be classified into two types: the passive setup with only one intensity, and the active device with more than one intensity. For those active device, implementing two-, three- or four-intensity decoy states^17^,^18^,^19^,^20^,^21^,^22^,^23^,^24^,^25^, where in real-life, an acousto- or electro-optic modulator is often used to switch between different decoy states with high speed, they will inevitably result in intensity uncertainty during parameter estimations, and thus deteriorate their practical performance. For passive setups^28^, one has to do the worst-case parameter estimation on the contributions from two-single-photon pulses owning to very few input parameters. Here, we will present a new scheme on implementing the MDI-QKD protocol with HSPS while using only one-intensity decoy state. In this scheme, we record all the successful detection events and mark them with different tags by classifying the heralding photons into different species, so that we can possess many input parameters and carry out very accurate estimations for the two-single-photon pulse contributions.

The paper is organized as follow: At the beginning, we present the core idea on how to generate the passive heralded single-photon sources; Second, we propose to implement the passive heralded single-photon sources into the MDI-QKD; Third, we carry out corresponding numerical simulations and compare its performance with other often used decoy-state proposals, e.g., the standard three-intensity decoy-state MDI-QKD using either the HSPS or the WCS. Finally, a summary and outlook are given at the end of the paper.

## The passive heralded single-photon sources

Normally, the HSPS can be generated from the parametric down-conversation (PDC) process, which can be either a thermal or poissonian distribution^12^. For simplicity, here we use the poissonian distributed PDC source as an example to describe the scheme. (In the case of thermal distribution, it will show similar behavior). The PDC process can generate a squeezed two-mode field, each denoted as the idler mode (I mode) and the signal mode (S mode) individually. The two-mode field can be written as: 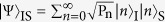, where |*n*〉 represents an *n*-photon state, 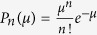, and *μ* is the average photon number per time slot.

In most former HSPS schemes, the idler mode is often locally detected with a photon diode at the sender’s side, and the signal mode is encoded with useful information and sent to the receiver through the quantum channel. Meanwhile, the sender delivers a synchronization signal to the receiver whenever the local phot-diode clicks. This is the so-called HSPS. However, below we will configure the devices in a different manner.

**Figure 1:**
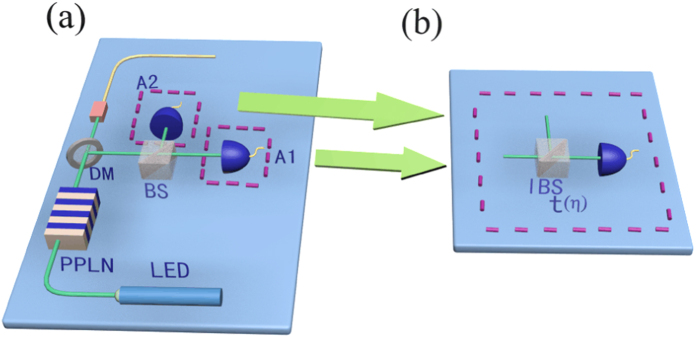
(**a**) The schematic setup of generating the passive HSPS. The pump light is generated from a light emitting diode (LED). After passing though the Periodically Poled Lithium Niobate (PPLN) crystal, the parametric down-conversion photon pairs (idler and signal) are separated by a dichroic mirror (DM). The idler mode is split into two parts by a beam-splitter (BS) and sent into two single-photon detectors (*A*_1_ and *A*_2_) respectively. (**b**) The illustration of single-photon detection in the idler mode, where an imaginary beam-splitter (IBS) is positioned before each local detector. *t* is the IBS transmissivity.

The schematic setup of our new scheme on generating the passive HSPS is shown in Fig. 1, where the most important change is to split the idler mode into two paths and then send each into a local single-photon detector (*A*_1_ and *A*_2_) separately. In all, the click events in the two local detectors may consist of four kinds of possibilities, each denoted as *X*_*i*_ (*i* = 1, 2, 3, 4): (1) Non-clicking; (2) Only one clicking at *A*_1_; (3) Only one clicking at *A*_2_; (4) Clicking at both *A*_1_ and *A*_2_.

We define 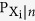 as the probability of the *X*_*i*_ events occurring if given an *n*-photon state in the idler mode. Then the signal state will be projected into 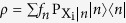 (un-normalized), where *f*_*n*_ is the photon-number distribution in the S mode. In the following, let’s derive the construction of the *X*_*i*_ event. To simplify the description, let’s begin with perfect detector efficiency for *A*_1_ and *A*_2_, and we will deal a bit later in the manuscript with non-unity detector efficiency by assuming “imaginary beam splitters”.

First, we denote 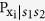 as the probability of the *X*_*i*_ event occurring given a 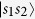 projection state. For a vacuum projection state, the corresponding local detector will click with a probability of *d*_*i*_ (the dark count rate), and non-clicking with a probability of (1 − *d*_*i*_). While for a non-vacuum projection state, the local detector will surely click with 100% probability. We can then list all the probabilities of the four (*X*_*i*_) events taking place as shown in Table 1.

Second, we define 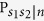 as the probability of projecting an *n*-photon state into state 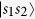. For an *n*-photon number state, after passing through a beam-splitter (BS) in the idler mode, it is changed into:


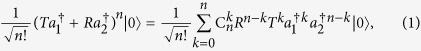


where the right side of the equation follows a binomial distribution, and 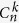 is the binomial coefficient, defined as 
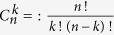
; *T*^2^ represents the transmission efficiency of the BS, denoted as *t*; *R*^2^ corresponds to the reflection efficiency, denoted as (1 − *t*).

As illustrated in Fig. 1, after the first BS we combine the coupling efficiency and detection efficiency in each path, and treat it as the transmission efficiency (*η*_*i*_, *i* = 1, 2) of an imaginary beam-splitter (IBS), and the loss corresponds to the reflection efficiency (1 − *η*_*i*_). After passing through the two IBSs, only the transmitted photons are collected. Now the state can be expressed as:


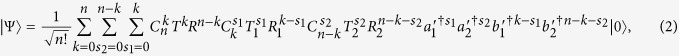


where 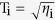, and 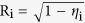, (*i* = 1, 2).

Then we can get the corresponding projection probability as:


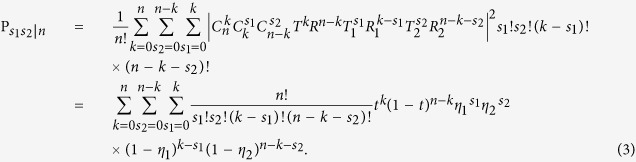


For any input *n*-photon state, the probability of occurring the *X*_*i*_ heralding event can be written as:


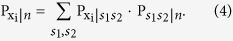


The corresponding heralded signal state is :


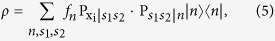


where the analysis of 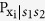 can be found in Table 1, and 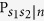 has been formulated in Eq. (3). Now with the above, we can do the calculation for any *X*_*i*_ event and any quantum efficiency.

In the following, we will denote the above *X*_1_, *X*_2_ and *X*_3_ events as the *x*, *y* and *z* state respectively. Correspondingly, in the photon-number space, we have 
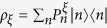
, with 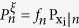, (*ξ* = *x*, *y*, *z*).

According to Eqs (1, 2, 3, 4, 5), we get the simplified photon-number distribution for the *x*, *y* and *z* state as


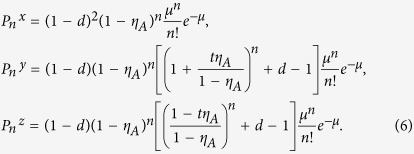


Here for simplicity we have assumed that all the local detectors have the same dark count rate, i.e., *d*_*i*_ = *d*. Besides, we reasonably set 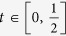, and *η*_*A*_ ∈ [0, 1].

For any 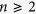, we find


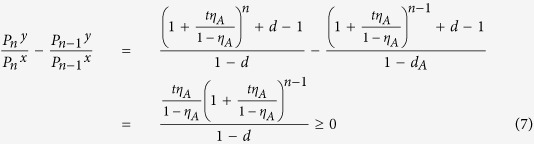


and


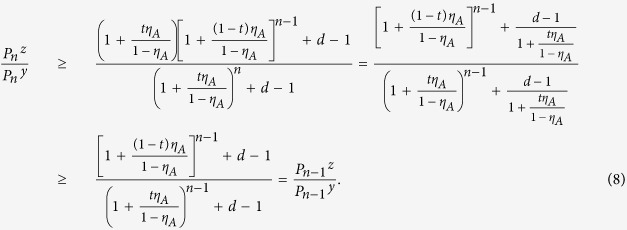


With the conditions above, we get the following inequalities:


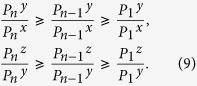


For further discussion, we define another useful quantity 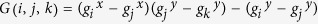 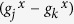 where 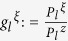, *ξ* = *x*, *y*, *z*, 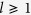.

For any 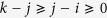, we can demonstrate


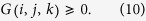


See the "Proof" section for the detailed proof. With the two inequalities, one can directly apply Wang’s formulas for the yield and phase-flip rate of single-photon pairs in ref. 25.

## Implementing the passive HSPS into the MDI-QKD

**Figure 2:**
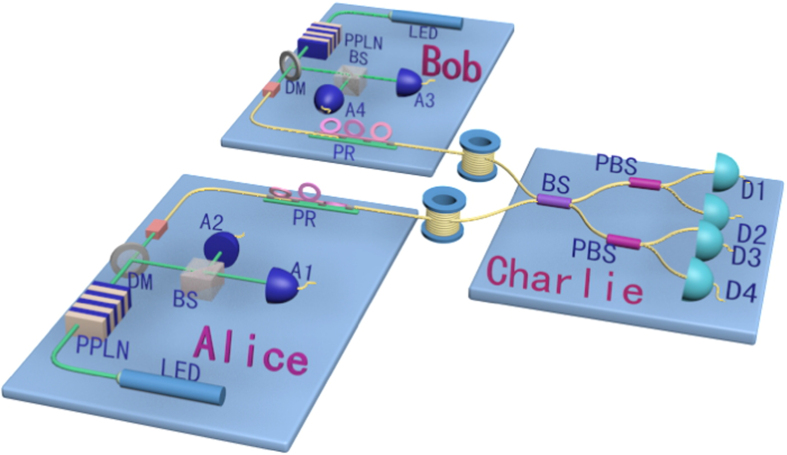
The schematic setup of our new passive-decoy state MDI-QKD protocol. Alice and Bob generate the passive HSPS as illustrated in Fig. 1, and randomly code each signal pulse into one of the four polarization states (horizontal (*H*), vertical (*V*), 45 degrees (+) and 135 degrees (−)) with a polarization rotator (PR). Then they simultaneously send their signal pulses to the third party (Charlie) through the quantum channel. Charlie apply a partial Bell-state projection measurement on pulses from both Alice and Bob. *A*_*i*_ (*i* = 1, 2, 3, 4): triggering single-photon detectors. *D*_*i*_ (*i* = 1, 2, 3, 4): triggered single-photon detectors. PBS: polarization beam-splitter. BS: beam-splitter.

The MDI-QKD was designed to remove all possible side-channel attacks and show attractive performance in real-life implementation. In MDI-QKD, both Alice and Bob send signals to an untrusted third party (UTP), Charlie. After a Bell state measurement, Charlie announces whether the measurement is successful. Then the successful event will be employed for key distribution. In order to make the MDI-QKD more practical, usually a decoy-state method is implemented in parallel. In most other schemes, it requires both Alice and Bob to randomly modulate their signal light into different intensities, and then do estimations with corresponding successful events. While here in our new scheme, only one-intensity signal light is applied at either Alice or Bob’s side, and then process parameter estimations by considering different counting events conditional on case *X*_*i*_ (*i* = 1, 2, 3, i.e., *x*, *y* and *z* state) as introduced above. The schematic experimental setup of the scheme is shown in Fig. 2.

In fact, the security of our proposal is equivalent to the processes as following: First, both Alice and Bob send out all heralded signal pulses (signal mode), and the UTP records all the successful counting events by do projecting measurement; Second, Alice and Bob start to send out heralding signals from local detectors, and correspondingly the UTP can divide all the successful counting events into different species (signal states or decoy states) and marked with different tags; Third, the UTP announce the tags of each successful event, and the legitimate users apply corresponding bit-flip operations and get the raw keys; Moreover, error correction and privacy amplification processes are carried out; Finally, people carry out parameter estimation processes. From the above, we find that during the signal transmission Eve is unable to judge which is the signal state and which is the decoy state, and has to apply the same attack strategy on all the pulses (signal state and decoy state), and his eavesdropping will certainly be discovered by the legitimate users by error tests.

In this scheme, for simplicity, we assume both Alice and Bob possess the same passive setup for signal generation. Then each of them can send out signals with *x*, *y* and *z* state individually. Whenever Alice sends out an *α* state and Bob sends out a *β*, with *α*, *β* ∈ (*x*, *y*, *z*), the average counting rate (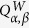) and the mean quantum-bit errors 
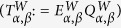
 can be written as:


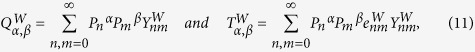


where we label W as the Z- or X-basis. The Z- or X-basis can be considered independently, hereafter we shall therefore omit the superscript W without causing any confusion. The subscripts *n*, *m* each represents the numbers of the photons sent by Alice or Bob respectively. 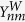 and 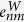 each corresponds to the conditional yield or the error rate when Alice sends an *n*-photon state and Bob sends an *m*-photon state. 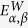 denotes the average quantum-bit error-rate.

Below we define


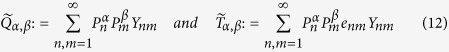


with


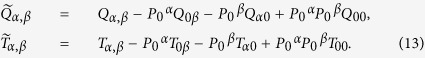


According to Wang *et al*.’s work in ref. 25, once the source states satisfy the inequalities in (9) and (10), we can immediately get the lower-bound of the counting rate for the two-single-photon pulses (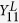) as


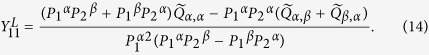


Similarly, we can get the upper bound of the quantum-bit error-rate for the two-single-photon pulses (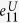)^25^:


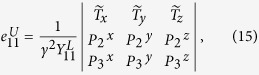


where 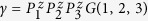, for *ξ* ∈ (*x*, *y*, *z*), 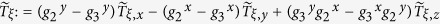.

In the new scheme, the Z-basis is used for key generation, and the X-basis is for error testing. From Eqs (14) and (15), we can obtain the lower-bound for the yield of two-single-photon pulses in the Z basis (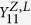) and the upper-bound for the quantum-bit error-rate of two-single-photon pulses in the X basis (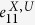). Moreover, the average counting rate and the mean quantum-bit error-rate can be observed experimentally. With all the above, we can calculate the secure key generation rate with the following formula:


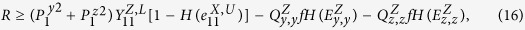


where *f* is the error correction efficiency, and here we take *f* = 1.16^16^,^18^; 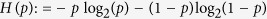. To simplify the calculation, we only use the *y* and *z* state for the key distillation. In fact, all the four kinds of events (*X*_*i*_, *i* = 1, 2, 3, 4) can be used to distill the final keys. That means the performance of the new scheme should be even better when taking all the successful events into consideration.

## Numerical simulation

With the formulae above, we can perform a numerical simulation for our new passive scheme on the decoy-state MDI-QKD, and further compare its performance with other practical methods, e.g. the conventional three-intensity decoy-state MDI-QKD using either WCS or HSPS^17^,^22^. In real-life experiment, the average gains and the average quantum-bit error-rates can be directly measured. While in numerical simulations, we should use a reasonable model to predict what *should probably be observed* in experiment. By referring the linear model in ref. 19, we can give a prediction for the *probably observed* values of the gains and the quantum-bit error-rates. For fair comparison, we assume the same parameters as in refs 16 and 18 in our simulation, see Table 2.

**Figure 3:**
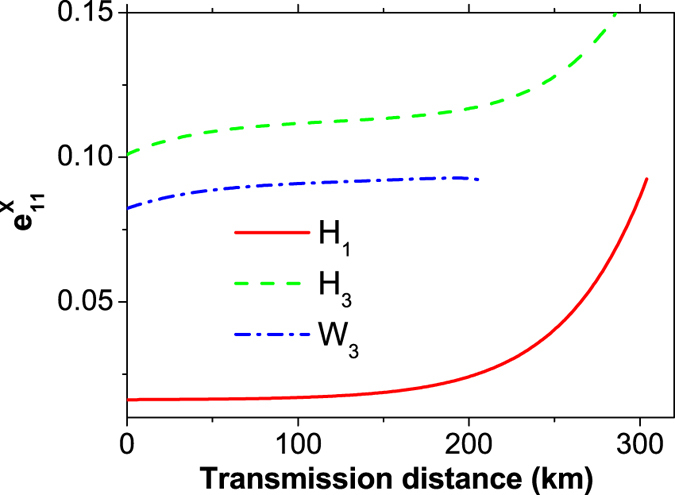
Comparison of the estimation value of 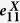 between different methods. The solid line (*H*_1_) refers to our new scheme, the dashed line (*H*_3_) represents the standard three-intensity decoy-state MDI-QKD using HSPS, and the dash-dotted line (*W*_3_) corresponds to the case of using conventional three-intensity decoy-state MDI-QKD using WCS. For simplicity, we set the intensity of the decoy state as *v* = 0.1 for *H*_3_ and *W*_3_.

**Figure 4:**
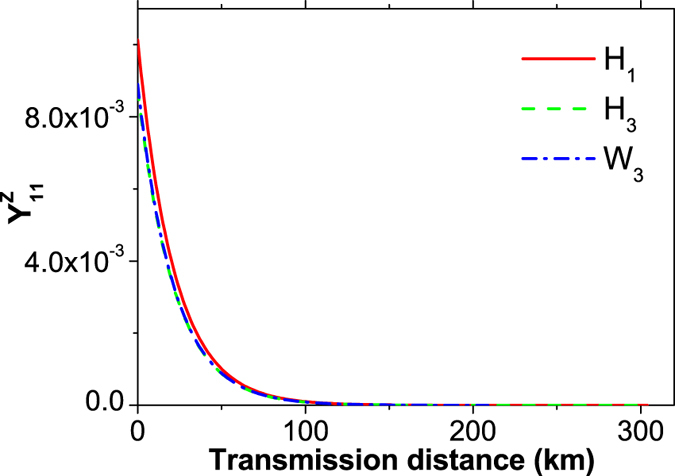
Comparison of the estimation value of 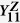 between different methods. The solid line (*H*_1_) refers to our new scheme, the dashed line (*H*_3_) represents the standard three-intensity decoy-state MDI-QKD using HSPS, and the dash-dotted line (*W*_3_) corresponds to the case of using conventional three-intensity decoy-state MDI-QKD using WCS. For simplicity, we set the intensity of the decoy state as *v* = 0.1 for *H*_3_ and *W*_3_.

**Figure 5:**
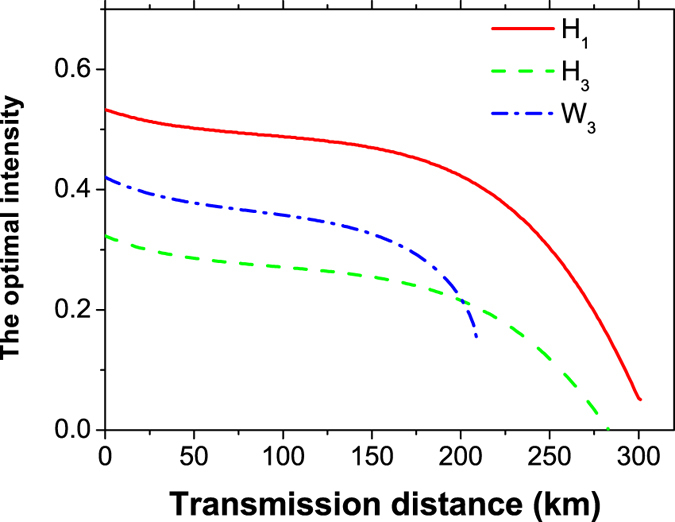
Comparison of the optimal intensity of the signal state *u* between different methods. The solid line (*H*_1_) refers to our new scheme, the dashed line (*H*_3_) represents the standard three-intensity decoy-state MDI-QKD using HSPS, and the dash-dotted line (*W*_3_) corresponds to the case of using conventional three-intensity decoy-state MDI-QKD using WCS. For simplicity, we set the intensity of the decoy state as *v* = 0.1 for *H*_3_ and *W*_3_.

**Figure 6:**
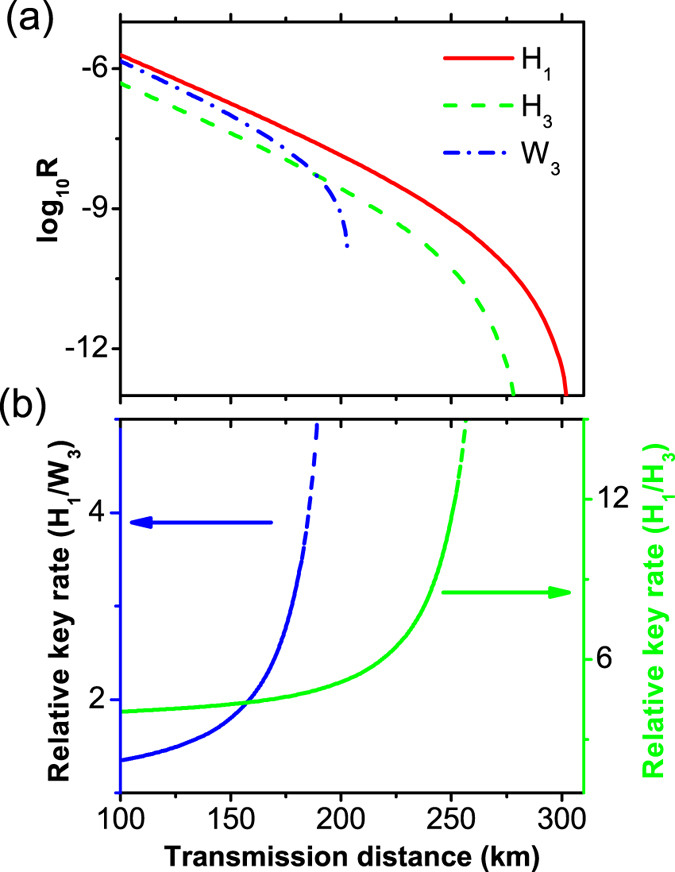
(**a**) Comparison of the final key generation rate *R* between different methods. The solid line (*H*_1_) refers to our new scheme, the dashed line (*H*_3_) corresponds to the standard three-intensity scheme using HSPS, and the dash-dotted line (*W*_3_) represents a conventional three-intensity MDI-QKD using WCS. (**b**) The ratio between the key generation rates of the new proposed passive scheme and the other two, see the left and right axes respectively.

In the source generation part, the non-degenerate parametric down-conversion process is often used to generate non-degenerated photon pairs, e.g., one is within the telecommunication wavelength range suitable for fiber transmission, and the other is within the visible wavelength range, convenient for detection. Therefore, it is reasonable to assume the local detectors with a detection efficiency of 75%, and a dark count rate of 10^−6^ (commercial products SPCM-NIR-16 or SPCM-AQRH-16 APD)^25^. Corresponding simulation results have been displayed in Figs 3, 4, 5 and 6.

In Fig. 3, we compare the estimation value for the quantum-bit error-rate of two-single-photon pulses (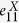) among our new scheme (*H*_1_), the conventional three-intensity decoy-state MDI-QKD using HSPSs (*H*_3_)^22^ and the standard three-intensity decoy-state MDI-QKD using WCSs (*W*_3_)^17^. We can find from Fig. 3 that, our new scheme shows significantly lower bound of the *e*_11_ than the other two schemes, which is on one hand due to the many kinds of successful counting events, and on the other hand owning to the usage of the newest estimating formula, Eq. (15).

The comparison of *Y*_11_ in the Z basis between the above three methods is shown in Fig. 4. The conventional three-intensity decoy-state MDI-QKD using the HSPS (*H*_3_) and using the WCS (*W*_3_) shows a similar level of 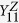. In contrast to them, the new proposed passive MDI-QKD (*H*_1_) obviously exhibits higher values.

In Fig. 5, we show a comparison for the optimal intensity of the signal state (*μ*) for different kinds of methods. Compared with the other two lines (*W*_3_ and *H*_3_), our new passive scheme (*H*_1_) presents superior values from the beginning to the end.

Moreover, we show a comparison for the key generation rate (*R*) for our new passive scheme(*H*_1_), for the standard three-intensity decoy-state MDI-QKD using HSPSs (*H*_3_), and for the conventional three-intensity decoy-state MDI-QKD using WCSs (*W*_3_), see Fig. 6(a). Compared with the other two proposals, the performance of our new scheme has drastically improved both the transmission distance and the final key generation rate. For a more vivid comparison, we also calculate the relative key generation rate between our new passive scheme and the other two three-intensity decoy-state methods, see the left and right axes in Fig. 6(b), respectively. We find that compared with the standard three-intensity decoy-state MDI-QKD using HSPSs, our new passive scheme can obtain more than five times enhancement in the key generation rate at long distances (>200 *km*). While compared with the conventional three-intensity decoy-state MDI-QKD using WCSs, it can exhibit more than 100% enhancement in the key generation rate, and achieve more than 100 *km* longer transmission distance.

## Conclusion

In summary, we have introduced a new protocol for the measurement-device-independent quantum-key-distribution with heralded single-photon sources involving only one-intensity decoy state. The key features are: At the source generation part, we split the triggering signals and send into different local detectors. By recording different kinds of detection events in the local detectors, we can divide the triggered events into different species at the receiver’s side. Moreover, during parameter estimations, we have implemented the newest formulae, i.e., Eqs (14) and (15) to give an upper or lower bound for the counting rate and the quantum-bit error-rate of two-single-photon pulses. Consequently, we obtain many input parameters and can do very accurate estimations for the two-single-photon pulse contributions. Furthermore, by carry out corresponding numerical calculations, we compare the new scheme with other often used three-intensity decoy-state methods, demonstrating that the new proposed approach could exhibit outstanding performance among those compared.

Besides, we should declare that if we take the source errors into consideration, the new proposed passive scheme will exhibit even predominant capability than those active decoy-state methods. Because no intensity modulator is applied in our new scheme, and thus avoids source uncertainties. These unfortunately exist in other two-, three-, or four-intensity decoy-state methods. Therefore, it may be a promising candidate for the implementation of quantum key distribution in the near future.

In addition, we have noted that recently a new novel four-intensity decoy-state protocol [Phys. Rev. A 93, 042324 (2016)] have been proposed by Wang *et al*.^29^, which shows excellent performance when accounting for statistical fluctuation and using biased basis. It should be interesting to implement their method into our present passive scheme which deserves further study in our future research.

## Proof

In order to demonstrate inequality (10), which states that 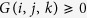, when 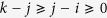, we recall that the Vandermonde determinant defined as


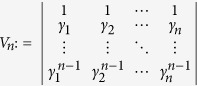


satisfies *V*_*n*_ > 0 for any 0 < *γ*_1_ < *γ*_2_ < ··· < *γ*_*n*_, which follows from *V*_*n*_ = Π_*i*<*j*_(*γ*_*j*_ − *γ*_*i*_). Now we establish the result which will be used in deriving inequality (10): The generalized Vandermonde determinant defined as


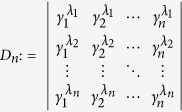


satisfies *D*_*n*_ > 0 for any 0 < *γ*_1_ < *γ*_2_ < ··· < *γ*_*n*_ and 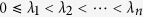.

To establish this, we proceed by induction method. First, it is clear that *D*_1_ > 0. Now assume that *D*_*n*−1_ > 0, we will show that *D*_*n*_ > 0. Note that


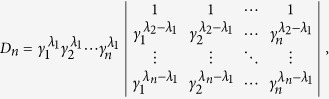


in order to show that *D*_*n*_ > 0, we may assume without loss of generality that *λ*_1_ = 0, and only consider *D*_*n*_ of the following form


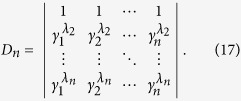


We will show that *D*_*n*_ > 0 for 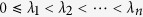.

First, consider *D*_*n*_ as a function of *γ*_*n*_ and note that 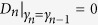. If we can show that 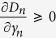 for *γ*_*n*_ > *γ*_*n*−1_, then we are done. Consider 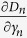 as a function of *γ*_*n*−1_, we have 
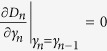
. Thus it suffices to show that 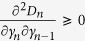 for *γ*_*n*−1_ > *γ*_*n*−2_. If one proceeds similarly, it suffices to show that


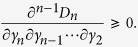


From Eq. (17) it is clear that


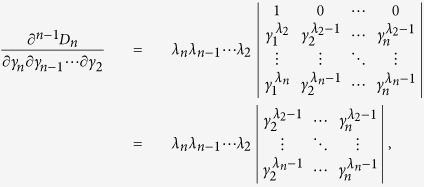


which is positive by assumption.

With the above preparation, we proceed to prove inequality (10). As introduced above, in the new passive decoy-state scheme, Bob’s counting events can be divided into four species by conditioning them on Alice’s heralding events *X*_*i*_, (*i* = 1, 2, 3 and 4). The first three have been denoted as states *x*, *y* and *z* respectively, and their photon-number distribution can be written as in Eq. (6). By substituting Eq. (6) into condition (10), we obtain


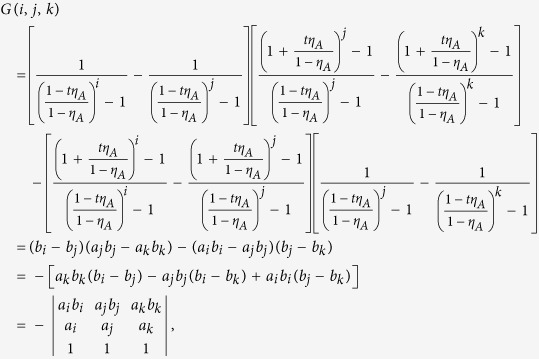


where 
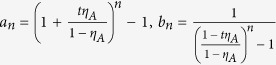
. In order to show 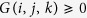, it suffices to show that


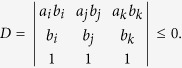


We denote 
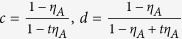
, so 
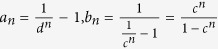
.

Note that


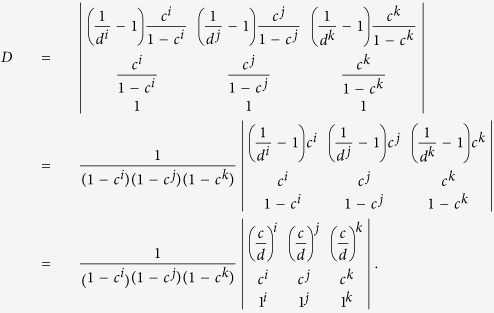


Due to the 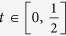, we can get 0 < *c* < *d*, and 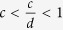. Thus,


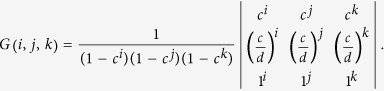


By the property of the generalized Vandermonde determinant inequality, the above expression is non-positive. It completes the proof of inequality (10).

## Additional Information

**How to cite this article**: Wang, Q. *et al*. Realizing the measure-device-independent quantum-key-distribution with passive heralded-single photon sources. *Sci. Rep.* **6**, 35394; doi: 10.1038/srep35394 (2016).

## Figures and Tables

**Table 1:** Probability of the (*X*_*i*_) event occurring.

Case	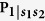	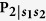	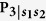	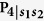
*X*_1_ : *s*_1_ = 0, *s*_2_ = 0	(1 − *d*_1_)(1 − *d*_2_)	*d*_1_(1 − *d*_2_)	*d*_2_(1 − *d*_1_)	*d*_1_*d*_2_
*X*_2_ : *s*_1_ ≠ 0, *s*_2_ = 0	0	(1 − *d*_2_)	0	*d*_2_
*X*_3_ : *s*_1_ = 0, *s*_2_ ≠ 0	0	0	(1 − *d*_1_)	*d*_1_
*X*_4_ : *s*_1_ ≠ 0, *s*_2_ ≠ 0	0	0	0	1

**Table 2:** Parameters values for simulations.

*η*_*C*_	*d*_*C*_	*e*_*d*_	*e*0	*γ*
14.5%	3.0 × 10^−6^	1.5%	0.5	0.2 dB/km

*η*_*C*_ and *d*_*C*_ are the detection efficiency and dark count rate at the UTP’s side; *e*_*d*_ is the probability that the survived photon hits the wrong detector, which is independent of the transmission distance, and *e*_0_ is the error rate of dark count; *γ* is the channel loss constant.

## References

[b1] Bennett C. H. & Brassard G. Quantum cryptography: Public key distribution and coin tossing. Proc. of IEEE Int. Conf. on Computers, Systems, and Signal Processing [175–179] (IEEE, New York, 1984).

[b2] Lo H. K. & Chau H. F. Unconditional security of quantum key distribution over arbitrarily long distances. Science 283, 2050–2056 (1999). 10092221 10.1126/science.283.5410.2050

[b3] Shor P. W. & Preskill J. Simple proof of security of the BB84 quantum key distribution protocol. Phys. Rev. Lett. 85, 441–444 (2000). 10991303 10.1103/PhysRevLett.85.441

[b4] Mayers D. Unconditional security in quantum cryptography. J. ACM 48, 351–406 (2001).

[b5] Brassard G., Lütkenhaus N., Mor T. & Sanders B. C. Limitations on practical quantum cryptography. Phys. Rev. Lett. 85, 1330–1333 (2000). 10991544 10.1103/PhysRevLett.85.1330

[b6] Lütkenhaus N. Security against individual attacks for realistic quantum key distribution. Phys. Rev. A 61, 052304 (2000).

[b7] Lütkenhaus N. & Jahma M. Quantum key distribution with realistic states: photon-number statistics in the photon-number splitting attack. New J. Phys. 4, 44.1–44.9 (2002).

[b8] Hwang W. Y. Quantum key distribution with high loss: Toward global secure communication. Phys. Rev. Lett. 91, 057901 (2003). 12906634 10.1103/PhysRevLett.91.057901

[b9] Wang X.-B. Beating the photon-number-splitting attack in practical quantum cryptography. Phys. Rev. Lett. 94, 230503 (2005). 16090451 10.1103/PhysRevLett.94.230503

[b10] Lo H.-K., Ma X. F. & Chen K. Decoy state quantum key distribution. Phys. Rev. Lett. 94, 230504 (2005). 16090452 10.1103/PhysRevLett.94.230504

[b11] Wang Q., Wang X.-B. & Guo G.-C. Practical decoy-state method in quantum key distribution with a heralded single-photon source. Phys. Rev. A 75, 012312 (2007).

[b12] Wang Q. & Karlsson A. Performance enhancement of a decoy-state quantum key distribution using a conditionally prepared down-conversion source in the Poisson distribution. Phys. Rev. A 76, 014309 (2007).

[b13] Wang Q., Wang X.-B., Bjork G. & Karlsson A. Improved practical decoy state method in quantum key distribution with parametric down-conversion source. Europhysics Letters 79, 4 (2007).

[b14] Wang Q. . Experimental decoy-state quantum key distribution with a sub-Poissionian heralded single-photon source. Phys. Rev. Lett. 100, 090501 (2008). 18352685 10.1103/PhysRevLett.100.090501

[b15] Braunstein S. L. & Pirandola S. Side-Channel-Free Quantum Key Distribution. Phys. Rev. Lett. 108, 130502 (2012). 22540685 10.1103/PhysRevLett.108.130502

[b16] Lo H.-K., Curty M. & Qi B. Measurement-device-independent quantum key distribution. Phys. Rev. Lett. 108, 130503 (2012). 22540686 10.1103/PhysRevLett.108.130503

[b17] Wang X.-B. Three-intensity decoy-state method for device-independent quantum key distribution with basis-dependent errors. Phys. Rev. A 87, 012320 (2013).

[b18] Wang Q. & Wang X.-B. Efficient implementation of the decoy-state measurement-device-independent quantum key distribution with heralded single-photon sources. Phys. Rev. A 88, 052332 (2013).

[b19] Wang Q. & Wang X.-B. Simulating of the measurement-device-independent quantum key distribution with phase randomized general sources. Sci. Rep. 4, 04612 (2014). 10.1038/srep04612 PMC3985083 24728000

[b20] Wang D. . Quantum key distribution with the single-photon-added coherent source. Phys. Rev. A 90, 062315 (2014).

[b21] Wang D., Li M., Guo G.-C. & Wang Q. An improved scheme on decoy-state method for measurementdevice-independent quantum key distribution. Sci. Rep. 5, 15130 (2015). 26463580 10.1038/srep15130 PMC4604557

[b22] Zhu F. & Wang Q. The quantum key distribution based on heralded single photon source. Acta Optica Sinica 34, 0627002 (2014).

[b23] Ma X., Fung C.-H. F. & Razavi M. Statistical fluctuation analysis for measurement-device-independent quantum key distribution. Phys. Rev. A 86, 052305 (2012).

[b24] Yu Z.-W., Zhou Y.-H. & Wang X.-B. Three-intensity decoy-state method for measurement-device-independent quantum key distribution. Phys. Rev. A 88, 062339 (2013).

[b25] Zhou Y.-H., Yu Z.-W. & Wang X.-B. Tightened estimation can improve the key rate of measurement-device-independent quantum key distribution by more than 100%. Phys. Rev. A 89, 052325 (2014).

[b26] Tang Y.-L. . Measurement-Device-Independent Quantum Key Distribution over Untrustful Metropolitan Network. Phys. Rev. X 6, 011024 (2016).

[b27] Pirandola S. . High-rate measurement-device-independent quantum cryptography. Nature Photonics 9, 397 (2015).

[b28] Shan Y.-Z. . Measurement-device-independent quantum key distribution with a passive decoy-state method. Phys. Rev. A 90, 042334 (2014).

[b29] Zhou Y.-H., Yu Z.-W. & Wang X.-B. Making the decoy-state measurement-device-independent quantum key distribution practically useful. Phys. Rev. A 93, 042324 (2016).

